# Rapid and Quantitative Detection of TNF-α in Human Tears Using a Portable Electrochemiluminescence-Based Device

**DOI:** 10.3390/bios15100645

**Published:** 2025-09-29

**Authors:** Shaohong Qu, Boyu Zhu, Zihao Liu, Xing Chen, Peifang Dong, Lihang Zhu

**Affiliations:** 1 Nursing Department of Eye Center, The Second Affiliated Hospital, Zhejiang University School of Medicine, Hangzhou 310009, China; 2503118@zju.edu.cn; 2 Key Laboratory of Excited-State Materials of Zhejiang Province, Institute of Analytical Chemistry, Department of Chemistry, Zhejiang University, Hangzhou 310058, China; 12337013@zju.edu.cn; 3 Zhejiang Provincial Key Laboratory of Cardio-Cerebral Vascular Detection Technology and Medicinal Effectiveness Appraisal, Key Laboratory of Biomedical Engineering of Ministry of Education of China, Department of Biomedical Engineering, Zhejiang University, Hangzhou 310058, China; theolzao@zju.edu.cn (Z.L.); cnxingchen@zju.edu.cn (X.C.); 4 Department of Clinical Engineering, The Second Affiliated Hospital of Zhejiang University School of Medicine, Hangzhou 310009, China

**Keywords:** dry eye syndrome, tumor necrosis factor alpha, point-of-care testing, electrochemiluminescence, aptasensor

## Abstract

Personalized, point-of-care testing of human tears is essential for ocular disease diagnosis, yet it is hampered by picomolar biomarker levels and microliter sample volumes. In this work, we developed an integrated, portable electrochemiluminescence (ECL)-based device for rapid and quantitative detection of tumor necrosis factor alpha (TNF-α), a pivotal inflammatory marker in ocular surface disease, with particular relevance to dry eye syndrome (DES). The device integrates a miniaturized electrochemical cell for ECL reactions and a compact silica photomultiplier for signal measurement. A vertical silica mesochannel (VSM)-coated ITO electrode is also integrated and further functionalized with TNF-α-specific aptamers. The VSM enables the enrichment of ECL luminophores, thus enabling further amplification of ECL signals and enhancing sensitivity. A wide linear range from 0.1 to 200 pg/mL was achieved using 10-fold dilution of 3 μL tear samples. Overall, this study provides a portable, highly sensitive platform for personalized analysis of TNF-α in tear fluid, enabling rapid point-of-care assessment of DES.

## 1. Introduction

Human tears are a transparent, non-invasively accessible biofluid secreted primarily by the lacrimal glands [1,2]. Like sweat and saliva, tear fluid interfaces directly with the external environment while encapsulating a diverse array of biomolecules, establishing it as a promising diagnostic medium for clinical health monitoring [3]. This complex mixture, comprising electrolytes, proteins, lipids, metabolites, and signaling molecules such as cytokines, makes tear analysis a powerful tool for health assessment [4,5,6,7]. Among these biomarkers, the inflammatory cytokine tumor necrosis factor-alpha (TNF-α) is a key indicator for ocular surface disorders, particularly dry eye syndrome (DES) [8,9]. Elevated TNF-α levels in tears correlate highly with epithelial damage, symptom severity, and immune cell activation in DES [10].

However, tear-based cytokine quantification is challenged by biomarker concentrations in the picomolar range or below and sample volumes of only a few microliters [11]. Traditional enzyme-linked immunosorbent assays (ELISAs) achieve excellent sensitivity and specificity via enzymatic amplification [12]. However, their use in onsite clinics is hindered by their multi-step protocols involving incubation and washing, relatively large sample requirements, and reliance on benchtop instrumentation. Alternative approaches, such as label-free optical methods and miniaturized electrochemical sensors, mitigate some constraints, but suffer from complex optics, electrode fouling, and stability issues in biological matrices [13,14,15].

Electrochemiluminescence (ECL) is a light-emitting process induced by electrochemical reactions. Its discovery can be traced back to 1929 [16]. Over several decades, ECL has evolved from a laboratory phenomenon to a powerful analytical technique with high sensitivity and selectivity and broad applicability [17]. Particularly since the 1960s, advances in integrated circuits and highly sensitive photoelectric sensors have propelled ECL into a new stage with better understanding of mechanism and increasingly sophisticated detection equipment. In 1963, Kuwana et al. used pulsed voltage and a photomultiplier tube to investigate the ECL kinetics and mechanism of luminol [18]. In 1972, Bard et al. first observed the ECL of tris(2,2′-bipyridine) ruthenium (II) (Ru(bpy)_3_^2+^) in acetonitrile solution [19]. From then on, researchers started to explore the tris(2,2′-bipyridine) ruthenium (II) (Ru(bpy)_3_^2+^)/tri-n-propylamine (TPrA) co-reactant system and its derivatives, which came to be regarded as the cornerstone of ECL-based assays, and have been commercialized for in vitro diagnostics since the 1990s [20,21,22,23,24]. The elimination of external light excitation endows ECL with remarkable advantages, including a near-zero background, high sensitivity, and excellent spatiotemporal controllability. For instance, Dong et al. proposed an optical imaging approach for single-molecule reactions based on ECL, which enabled super-resolution microscopy [25]. Additionally, ECL has been demonstrated to be an ideal transduction technique for point-of-care testing due to its unique advantages of high spatiotemporal controllability and elimination of external light sources for excitation [26]. Most recently, Zhang et al. developed a portable ECL detection system integrated with a bipolar electrode chip for the simultaneous multiplexed detection of respiratory pathogens [27]. Compared with fluorescence (FL)-based detection, ECL inherently eliminates reliance on an external light source, contributing to reduced device complexity. Additionally, ECL signals can be regulated by electrochemical parameters, thus offering stable and reproducible signal output. These intrinsic features make ECL exceptionally well-suited to addressing the constraints of ultra-low analyte concentrations and severely limited tear volumes [28,29]. Integrating on-chip enrichment of ECL luminophores with sample dilution represents a compelling strategy for developing a portable, high-performance point-of-care testing (POCT) device for tear-based TNF-α monitoring.

**Figure 1:**
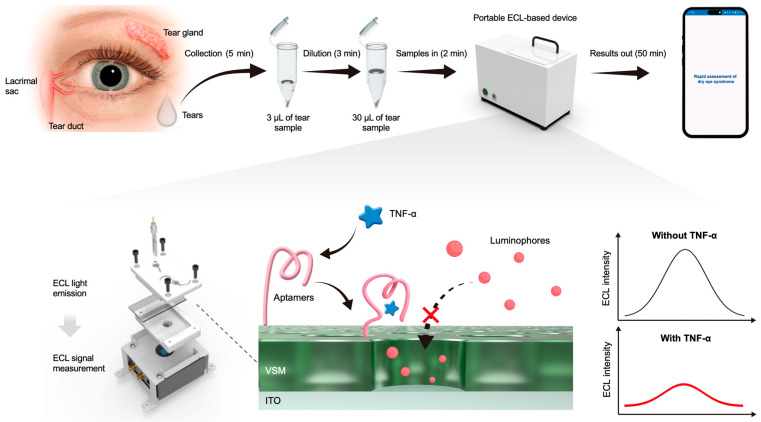
An illustration of the portable electrochemiluminescence (ECL)-based device designed to measure TNF-α for point-of-care assessment of dry eye syndrome (DES).

Herein, we present an integrated device designed to measure TNF-α for point-of-care assessment of DES progression. This device combines a miniaturized electrochemical cell featuring an indium tin oxide (ITO) electrode coated with vertical silica mesochannels (VSMs) and functionalized with tailored TNF-α-specific aptamers (Figure 1). Ru(dcpy)_3_^2+^ and TPrA were employed as the ECL luminophore and co-reactant, respectively. As a derivative of Ru(bpy)_3_^2+^, Ru(dcpy)_3_^2+^ introduces abundant carboxyl groups on the surface, thus offering enhanced chemical reactivity and biocompatibility while retaining the basic properties of Ru(bpy)_3_^2+^ [30,31,32,33]. The VSMs enhance the enrichment of ECL luminophores, thus allowing further amplification of the ECL signal and improved sensitivity. By measuring ECL signal attenuation, which is inversely correlated with the concentration of TNF-α, the device enables precise quantification of the target in diluted tear samples. Moreover, the device provides an automated analysis workflow, with results wirelessly transmitted to a smartphone for real-time monitoring. The design and fabrication of this device offer a highly efficient, portable solution for clinical evaluation of DES, showing great potential for POCT applications.

## 2. Materials and Methods

### 2.1. Chemicals and Materials

All chemicals and reagents were of analytical grade or higher and were used as received, without further purification. All aqueous solutions were prepared using ultrapure deionized water (18.2 MΩ cm). Sodium hydroxide (NaOH, >98%), hydrochloric acid (HCl, 37 wt%), acetone (>99%), ethanol (>99%), aqueous ammonia solution (28 wt%), tetraethyl orthosilicate (TEOS, >99%), hexadecyltrimethylammonium bromide (CTAB, >99%), tri-*n*-propylamine (TPrA, >98%), 3-glycidyloxypropyltrimethoxysilane (GPTMS, >97%), and bovine serum albumin (BSA, >98%) were purchased from Sigma-Aldrich (Shanghai, China). Bis (2,2′-bipyridyl) (4,4′-dicarboxyl-2,2′-bipyridyl) ruthenium (II) dichloride (Ru(dcpy)_3_Cl_2_, 97%) was purchased from SunaTech Inc (Suzhou, China). Indium tin oxide (ITO) electrodes (thickness of 100 nm, resistance of <15 Ω/square) were purchased from Kaivo Electronic Components (Zhuhai, China). Human TNF-α was purchased from Thermo Fisher Scientific (Waltham, MA, USA). The amino-modified TNF-α aptamer sequence (5′-GCGCCACTACAGGGGAGCTGCCATTCGAATAGGTGGGCCGC-3′) was purchased from Sangon Biotech (Shanghai, China) [34,35].

### 2.2. Preparation of the Electrodes

The VSM/ITO electrodes were prepared following the Stöber growth approach, as previously described [36,37,38,39]. First, the ITO electrodes were immersed in 1 M NaOH solution for 12 h. Subsequently, the electrodes were sequentially cleaned by ultrasonics in acetone, ethanol, and ultrapure deionized water. Next, the electrodes were immersed in a growth solution containing 30 mL ethanol, 70 mL deionized water, 10 μL aqueous ammonia solution, 80 μL TEOS, and 160 mg CTAB. The growth of VSM on the ITO electrodes was achieved by incubating the electrodes in a water bath at 60 °C for 20 h. The electrodes were then aged at 100 °C for 12 h, resulting in electrodes with surfactant micelles (SMs) inside the silica mesochannels. To remove the SMs from the mesochannels, the electrodes were immersed in 0.1 M HCl ethanol solution, and the VSM/ITO electrodes were thus obtained.

To functionalize the electrodes with aptamers, the electrodes were first immersed in a 3 mM GPTMS ethanol solution for 24 h at room temperature. Next, the electrodes were incubated with 50 μL of 1 μM aptamer solution for 2 h. It is noteworthy that the amount and density of the aptamer immobilized on the electrode surface could be controlled by changing the volume and concentration of the aptamer solution. After incubation, the electrodes were rinsed with 0.1 M PBS to remove unbonded aptamers. The resulting electrodes were then incubated in a 0.5% BSA solution to block non-specific adsorption sites, yielding the apt-VSM/ITO electrodes.

### 2.3. Fabrication of the Device

A portable device was fabricated for ECL measurement at the point of care. A silicon photomultiplier module (SiPM), purchased from Novel Device Laboratory (Beijing Normal University, Beijing, China), was integrated for quantification of ECL intensity. Additionally, a smartphone application was developed using Android Studio Flamingo (Google LLC, Mountain View, CA, USA). More details are presented in Section 3.1.

### 2.4. Detection of Tear Samples

This research was conducted in accordance with all of the ethical regulations approved by the Clinical Research Ethics Committee of the Second Affiliated Hospital, Zhejiang University School of Medicine (Ethic code:IR2025157). We confirm that all procedures involving human participants strictly adhere to the principles of the Declaration of Helsinki. Human tear samples were collected from three volunteers, following standard protocols. Specifically, tear samples were collected by a trained practitioner using sterile glass capillary tubes. The tip of the capillary tube was gently placed at the lower lateral canthus or the outer corner of the lower eyelid meniscus, without stimulation of reflex tearing. Then, tear samples were drawn into the tube via capillary action. Approximately 3 μL of tear samples were collected from each eye, which were immediately transferred to a sterile microcentrifuge tube for analysis. Notably, tear sample collection was performed without usage of any topical anesthetics to avoid contamination. For detection, the collected tear samples were firstly diluted 10-fold with ECL buffer. Then, the prepared electrode was incubated with the diluted tear samples. After incubation, the ECL reaction was triggered using CV with potentials ranging from 0 to 1.2 V and a scan rate of 100 mV/s. The concentration of TPrA was 10 mM, and the ECL signals of the electrodes were recorded by the device for subsequent analysis.

## 3. Results and Discussions

### 3.1. Design and Fabrication of the Device

**Figure 2:**
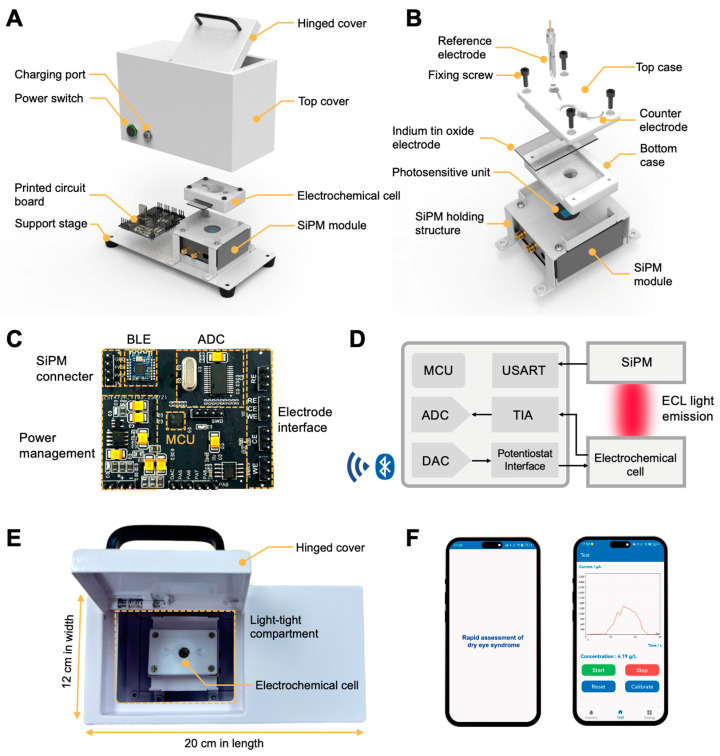
(**A**) An expanded view of the portable ECL-based device assembly. (**B**) Structural details of the electrochemical cell and SiPM module. (**C**) A photograph of the printed circuit board integrated within the device. (**D**) A block diagram of the portable ECL-based device. (**E**) A photograph of the assembled device. (**F**) A photograph of the developed smartphone application.

As shown in Figure 2A, the device consists of (i) a custom-designed miniaturized electrochemical cell for ECL reactions; (ii) a compact silicon photomultiplier (SiPM) module for accurate measurement of ECL signals; (iii) a printed circuit board (PCB) for data acquisition, processing, and wireless communication; and (iv) a high-capacity rechargeable lithium-ion battery for power. Notably, the SiPM module is vertically aligned beneath the electrochemical cell, thus allowing for highly efficient detection of ECL signals. The light emitted by the ECL reaction is transmitted through the transparent ITO electrode within the electrochemical cell and captured by the SiPM module.

As shown in Figure 2B, the electrochemical cell includes top and bottom cases made of polytetrafluoroethylene (PTFE), which can be joined together by fixing screws. To enable onsite and wireless detection of tear samples, a custom-designed PCB is incorporated within the device (Figure 2C). Figure 2D illustrates a circuit block diagram of the device. Various functional modules, including the microcontroller (MCU), the potentiostat module, the power management module, and the Bluetooth Low Energy (BLE) module, are integrated within the PCB. The device is designed to manage signal transduction and perform ECL tests. ECL signal data are wirelessly transmitted to a smartphone through the BLE module. The fully integrated device is stack-assembled with the above components, which are encapsulated with customized enclosures. The whole device measures 20 cm × 12 cm × 10 cm and weighs 1.8 kg, making it a promising platform for rapid and quantitative detection at the point of need (Figure 2E). Additionally, we developed an Android smartphone application to facilitate data collection, analysis, and visualization (Figure 2F).

### 3.2. Preparation and Characterization of the Electrodes

**Figure 3:**
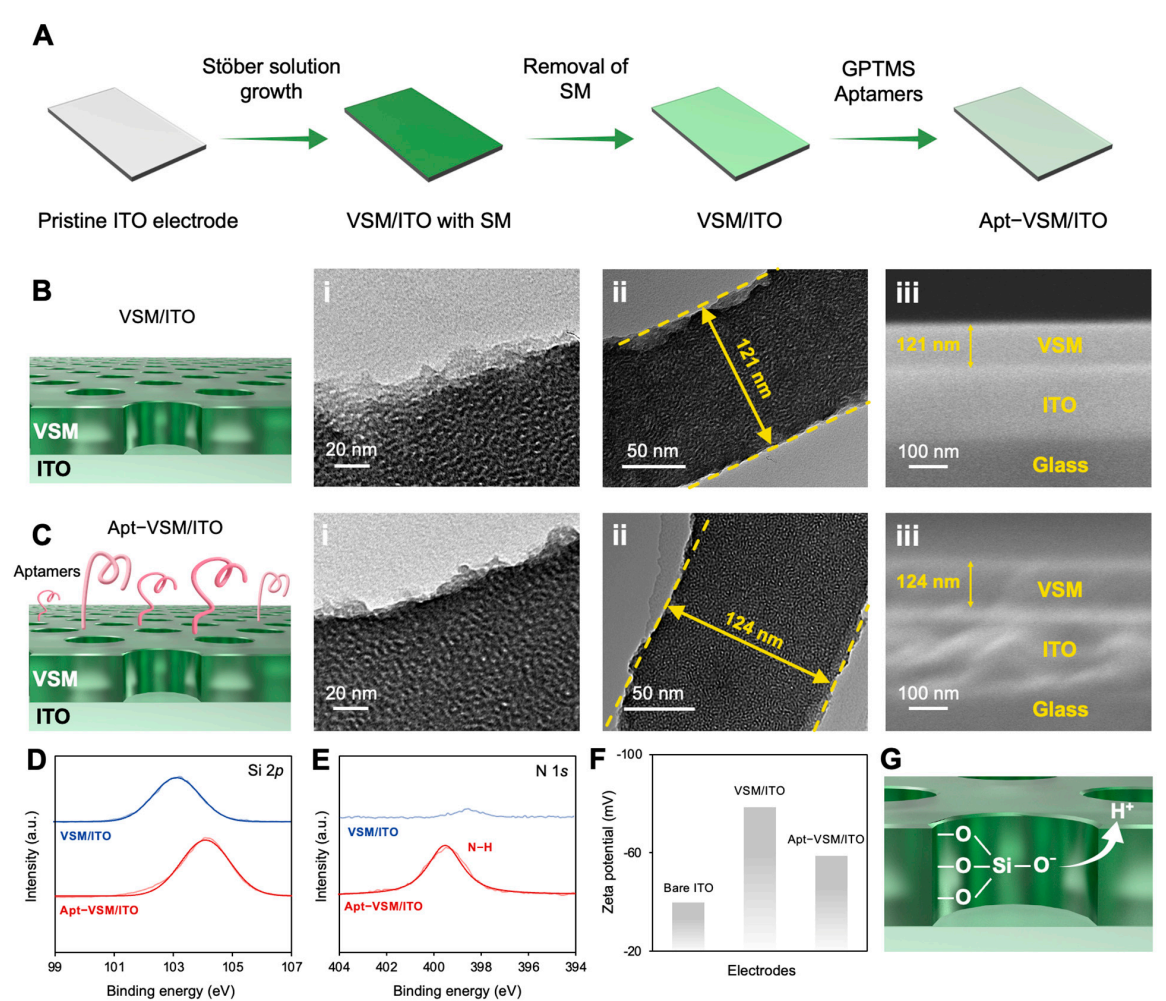
(**A**) A schematic illustration of the preparation of the VSM/ITO electrode and aptamer-functionalized VSM/ITO electrode (Apt-VSM/ITO). (**B**) TEM and SEM characterization of the VSM/ITO electrode. (**i**) Top-view TEM image of VSM. (**ii**) Cross-sectional TEM image of VSM. (**iii**) Cross-sectional SEM image of VSM/ITO electrode. (**C**) TEM and SEM characterization of the Apt-VSM/ITO electrode. (**i**) Top-view TEM image of Apt-VSM. (**ii**) Cross-sectional TEM image of Apt-VSM. (**iii**) Cross-sectional SEM image of Apt-VSM/ITO electrode. (**D**) High-resolution Si 2p spectra of the VSM/ITO and Apt-VSM/ITO electrodes. (**E**) High-resolution N 1s spectra of the VSM/ITO and Apt-VSM/ITO electrodes. (**F**) The zeta potential of the bare ITO, VSM/ITO, and Apt-VSM/ITO electrodes at pH 7. (**G**) A schematic illustration of VSM hydrolysis.

As shown in Figure 3A, the preparation of aptamer-modified VSM/ITO (Apt-VSM/ITO) involved the growth of VSMs and the construction of an aptamer-based recognition surface. Firstly, the VSMs were grown on a readily available ITO electrode using the Stöber solution method. After removing micelles, the surface of the VSM/ITO was functionalized with GPTMS. Specifically, the GPTMS reacted covalently with the silicon-hydroxyl groups exposed on the VSM/ITO, thus introducing epoxy groups onto the electrode surface. Amino-modified aptamers were covalently immobilized on the electrode surface as a recognition surface using an epoxy–amine reaction. Figure 3B illustrates TEM and SEM images of the VSM/ITO electrode, showing that the structure remained intact. The top-view TEM image in Figure 3B(i) shows the uniformly packed VSM channels with a pore diameter of approximately 2 to 3 nm. The cross-sectional-view TEM image in Figure 3B(ii) indicates that the VSMs coated on the ITO electrode were homogeneous, with a flat thickness of approximately 121 nm. The cross-sectional-view SEM image in Figure 3B iii confirms that VSMs grew on the ITO surface, and their thickness was consistent with that obtained in the TEM investigation. As shown in Figure 3C, the thickness of the Apt-VSM was approximately 124 nm, which can be ascribed to the modification with aptamers. Additionally, no significant structural changes were observed. Figure 3D compares the XPS spectra of VSM/ITO and Apt-VSM/ITO. As shown, the Si 2p characteristic peak positioned at 103.8 eV is clearly visible, suggesting the successful growth of VSMs. Additionally, a characteristic peak of Apt-VSM/ITO at 399.5 eV, corresponding to the N 1s, can be observed, proving the presence of amino groups on the VSMs in Apt-VSM/ITO [40]. We further characterized the electrodes by measuring their zeta potential. As shown in Figure 3E, the bare ITO electrode exhibited a negative zeta potential (−39.8 mV), suggesting a negatively charged surface. After the growth of VSMs, the zeta potential of the electrode shifted towards a more negative state, which may be ascribed to the abundant silanol groups on the surface and within the VSM channels (Figure 3F). Afterwards, the negative charge of the VSM/ITO changed from −78.5 mV to −58.7 mV following its amination and functionalization, indicating the successful preparation of Apt-VSM/ITO.

### 3.3. The Sensing Mechanism and Analytical Performance of the Device

**Figure 4:**
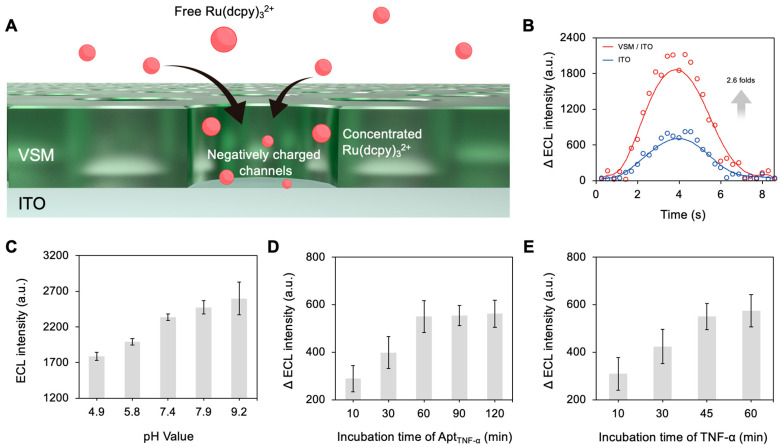
(**A**) A schematic illustration of ECL luminophore enrichment in VSMs. (**B**) The ECL signals of the bare ITO and VSM/ITO electrodes in 0.01 M PBS containing 50 μM of Ru(dcpy)_3_^2+^. (**C**) Optimization of pH. (**D**) Optimization of the incubation time for the TNF-α-specific aptamer. (**E**) Optimization of the incubation time for TNF-α.

For TNF-α detection, we selected the Ru(dcpy)_3_^2+^/TPrA co-reactant ECL system due to its superior emission efficiency, inherent stability, and well-established use in commercial diagnostics. As mentioned, the vertical silica mesochannels grown on ITO electrodes (VSM/ITO) form a negatively charged structure that electrostatically enriches the cationic Ru(dcpy)_3_^2+^ luminophore. Within the mesochannel scaffold, the cationic Ru(dcpy)_3_^2+^ luminophores are electrostatically attracted to the ITO surface (Figure 4A). Meanwhile, TPrA diffuses in and undergoes successive oxidation to radical cations and then neutral radicals. The oxidized Ru(dcpy)_3_^3+^ then reacts with ${TPrA}^{\cdot }$ to form the excited Ru(dcpy)_3_^2+^*, which later relaxes to the ground state with photon emission. Notably, the enrichment of Ru(dcpy)_3_^2+^ luminophores dramatically increases their local concentrations, yielding a substantially amplified ECL signal. As shown in Figure 4B, the ECL intensity achieved using the VSM/ITO electrode was approximately 2.6 times larger than that achieved using the bare ITO electrode. An essential condition associated with ECL analysis is the pH value. Before conducting quantification analysis, we optimized the assay pH by testing phosphate buffers from pH 4.9 to 9.2 (Figure 4C). As the pH increased, the VSM/ITO ECL intensity increased significantly, indicating more efficient deprotonation of TPrA under alkaline conditions. However, the signal became unstable above pH 7.9, likely due to hydrolysis of the VSM. Therefore, pH 7.4 was selected as the optimal condition for all subsequent assays. We further optimized the assay conditions, including the incubation time of the TNF-α-specific aptamer (Apt_TNF-α_) and the TNF-α. As shown in Figure 4D, Apt_TNF-α_ with an incubation time of 60 min gave the optimal results. The TNF-α incubation time was also optimized. Figure 4E confirms that the Apt_TNF-α_ and TNF-α completely bonded to form a stable complex within 45 min. Thus, this was selected as the optimal incubation time for TNF-α.

**Figure 5:**
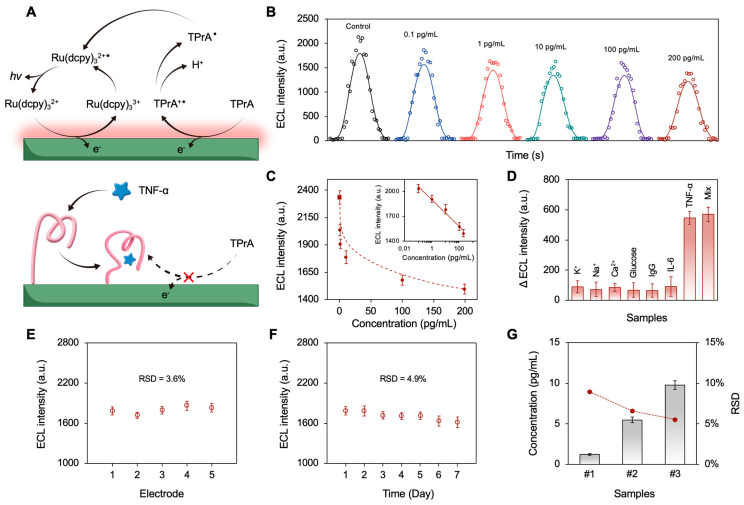
(**A**) Device sensing mechanism. (**B**) ECL responses of Apt-VSM/ITO electrodes with various concentrations of TNF-α. (**C**) Corresponding calibration curves. The inset shows the corresponding linear relationship between ECL intensity and the logarithm of target TNF-α. (**D**) Device specificity tests. (**E**) Device reproducibility tests. (**F**) Device storage stability tests. (**G**) Device recovery tests.

The analytical performance of the device was investigated under the optimized conditions. As illustrated in Figure 5A, a complex was formed upon specific binding of TNF-α to the aptamer immobilized on the VSM/ITO electrode, increasing interfacial steric hindrance. This steric hindrance impeded the diffusion of the co-reactant, TPrA, towards the electrode surface, thus attenuating the ECL intensity. Solutions with different TNF-α concentrations were prepared to test the sensitivity of the device. As shown in Figure 5B, the ECL intensity decreased with increasing the TNF-α concentrations ranging from 0.1 pg/mL to 200 pg/mL. As illustrated in Figure 5C, the ECL intensity exhibited a linear relationship with the logarithm of TNF-α concentration (from 0.1 pg/mL to 200 pg/mL). The regression equation was described by the formula y = −69.82 ln (x) + 1899.5, with a coefficient of determination (R^2^) of 0.98. According to the 3× signal-to-noise ratio (SNR) criterion, the limit of detection (LOD) was calculated to be 0.089 pg/mL. It is noteworthy that the calibration curve also revealed a dynamic detection range that spans the physiological levels of TNF-α in healthy subjects (<0.1 pg/mL) and DES patients (>100 pg/mL), even after tenfold dilution of tear samples. To evaluate the specificity of the proposed device, interference tests were conducted using common interfering substances in tears, including Na^+^(200 mM), K^+^(100 mM), Ca^2+^(1 mM), glucose (1 mM), IgG (1 μg/mL), and IL-6 (1 ng/mL). TNF-α was tested with a concentration of 10 ng/mL to simulate inflammatory conditions. As depicted in Figure 5D, negligible variation in ECL intensity was observed in the presence of interfering species, indicating high specificity. Reproducibility is another crucial factor for analysis, and was assessed using five parallel-prepared electrodes exposed to 10 pg/mL TNF-α, indicating a relative standard deviation (RSD) of 3.6% (Figure 5E). Long-term stability was also investigated by storing electrodes dry in a sealed container at 4 °C for 7 days. As shown in Figure 5F, no significant variation was observed during storage, with an RSD of 4.9%, indicating high storage stability of the device. Collectively, these results demonstrate significant potential for the developed device in practical TNF-α detection. Given that TNF-α was undetectable in tears from healthy individuals, performance was assessed using recovery tests. As shown in Figure 5G, diluted tear samples were spiked with TNF-α at concentrations of 1, 5, and 10 pg/mL, yielding satisfactory recoveries of between 98% and 102%. The RSD values from three repeat measurements were all below 10%, demonstrating the high sensitivity, stability, and reliability of the proposed device for clinical application in point-of-care diagnostics.

## 4. Conclusions

In summary, we designed and fabricated a portable ECL-based device that enables quantitative analysis of TNF-α from a minute volume of tears. Integration of a miniaturized electrochemical cell and a compact silica photomultiplier enabled the device to perform ECL measurements at the point of care. Additionally, a VSM-coated ITO electrode was prepared and further functionalized with TNF-α-specific aptamers. The electrode enabled the enrichment of ECL luminophores, enabling further amplification of the ECL signal and enhancing sensitivity. A broad linear dynamic range from 0.1 to 200 pg/mL was achieved with high specificity and stability. In addition, analytical accuracy in human tears was validated using recovery tests. We believe that this integrated and portable device will pave the way for the development of personalized and point-of-care devices for the diagnosis of ophthalmic diseases.

## Data Availability

The data presented in this study is available on request from the corresponding author.
